# Trisodium scandium bis­(orthoborate)

**DOI:** 10.1107/S1600536812015061

**Published:** 2012-04-25

**Authors:** Jinzhi Fang, Xinyuan Zhang, Jiyong Yao, Guochun Zhang, Kunpeng Wang

**Affiliations:** a National Center for Materials Service Safety, University of Science and Technology Beijing, Beijing 100083, People’s Republic of China; b Key Laboratory of Functional Crystal and Laser Technology, Technical Institute of Physics and Chemistry, Chinese Academy of Sciences, Beijing 100190, People’s Republic of China; c Graduate University of Chinese Academy of Sciences, Beijing 100049, People’s Republic of China

## Abstract

Single crystals of tris­odium scandium bis­(orthoborate), Na_3_Sc(BO_3_)_2_, have been obtained by spontaneous crystallization from an Na_2_O–Sc_2_O_3_–B_2_O_3_ melt. The crystal structure features a three-dimensional framework composed of planar [BO_3_]^3−^ groups and distorted ScO_6_ octa­hedra with Na atoms in the cavities. The Sc atom occupies a special position (Wyckoff position 2*b*, site symmetry -1) and of the two Na atoms, one occupies a special position (Wyckoff position 2*c*, site symmetry -1).

## Related literature

For Na_3_Sc_2_(BO_3_)_3_, see: Zhang *et al.* (2006 ▶) and for NaScB_2_O_5_, see: Becker & Held (2001 ▶). For similar structures, see: Cai *et al.* (2011 ▶). The program *STRUCTURE TIDY* (Gelato & Parthé, 1987 ▶) was used to standardize the structural data.

## Experimental

### 

#### Crystal data

Na_3_Sc(BO_3_)_2_
*M* *_r_* = 231.55
Monoclinic, 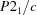

*a* = 5.0739 (10) Å
*b* = 8.9930 (18) Å
*c* = 7.029 (2) Å
β = 123.60 (2)°
*V* = 267.14 (11) Å^3^
*Z* = 2
Mo *K*α radiation
μ = 1.58 mm^−1^
*T* = 153 K
0.27 × 0.14 × 0.09 mm

#### Data collection

Rigaku Saturn724+ diffractometer
Absorption correction: multi-scan (*ABSCOR*; Higashi, 1995 ▶) *T*_min_ = 0.824, *T*_max_ = 1
3029 measured reflections
847 independent reflections
760 reflections with *I* > 2σ(*I*)
*R*_int_ = 0.026

#### Refinement

*R*[*F* ^2^ > 2σ(*F* ^2^)] = 0.027
*wR*(*F* ^2^) = 0.067
*S* = 1.22
847 reflections
58 parameters
Δρ_max_ = 0.35 e Å^−3^
Δρ_min_ = −0.62 e Å^−3^

### 

Data collection: *CrystalClear* (Rigaku, 2008 ▶); cell refinement: *CrystalClear*; data reduction: *CrystalClear*; program(s) used to solve structure: *SHELXS97* (Sheldrick, 2008 ▶); program(s) used to refine structure: *SHELXL97* (Sheldrick, 2008 ▶); molecular graphics: *DIAMOND* (Brandenburg, 2006 ▶); software used to prepare material for publication: *SHELXL97*.

## Supplementary Material

Crystal structure: contains datablock(s) I, global. DOI: 10.1107/S1600536812015061/fi2123sup1.cif

Structure factors: contains datablock(s) I. DOI: 10.1107/S1600536812015061/fi2123Isup2.hkl

Additional supplementary materials:  crystallographic information; 3D view; checkCIF report

## Figures and Tables

**Fig. 1.**
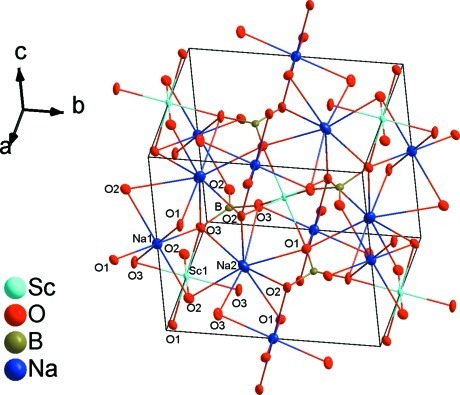
The structure of Na3Sc(BO3)2 with displacement ellipsoids drawn at the 80% probability level.

**Fig. 2.**
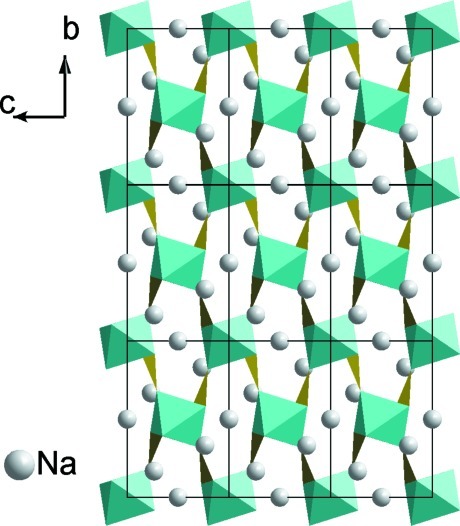
The structure of Na3Sc(BO3)2 given in the polyhedral description. [ScO6] octahedra are aqua, and [BO3] units are dark yellow.

## supplementary crystallographic information

### Comment

In efforts to identify new borates as optical materials or catalysts, investigations have been carried out in the Na_2_O—Sc_2_O_3_—B_2_O_3_ system, in which, two phases are already known, *viz*. Na_3_Sc_2_(BO_3_)_3_ (Zhang *et al.*, 2006) and NaScB_2_O_5_ (Becker & Held, 2001). Here, we report a new compound, Na_3_Sc(BO_3_)_2_.

The fundamental building units of the title compounds are triangular [BO_3_]^3-^ groups and irregular ScO_6_ octahedra. Each [BO_3_]^3-^ group connects to three ScO_6_ octahedra, and each ScO_6_ octahedron are connected to six [BO_3_]^3-^ groups by corner sharing, thereby constructing a three-dimensional framework whose cavities are filled with Na^+^ cations. Sc occupies a special position (Wyckoff position 2b, site symmetry –1), of the two Na atoms, one occupies a special positions (Wyckoff position 2c, site symmetry –1). Na2 occupies a general position. The B—O bond lengths range from 1.3708 (17) to 1.3802 (19) Å, and the mean O—B—O bond angles are equal to 120 (12)° which indicates that the [BO_3_]^3-^ groups are almost planar. The Sc^3+^ cation is six-coordinated by oxygen atoms to form a distorted ScO_6_ octahedron with Sc—O bond lengths ranging from 2.0765 (11) to 2.1285 (11) Å. The Na atoms appear in two crystallographically different environments. The Na1 and Na2 atoms are six- and eight-coordinated by oxygen atoms, respectively. The Na—O bond lengths range from 2.3448 (12) to 2.8515 (15) Å. These abovementioned values are normal in borates such as Na_3_Sc_2_(BO_3_)_3_ and NaScB_2_O_5_.

The structure of the title compound is closely related to Li_3_Sc(BO_3_)_3_ (Cai *et al.*, 2011) which is crystallized in the same space group. The crystal structure of Li_3_Sc(BO_3_)_3_ could also be characterized as a three-dimensional framework constructed by planar [BO_3_]^3-^ groups and irregular ScO_6_ octahedra. Both Na and Li atoms occupy two crystallographically independent sites, but Li atoms are four-coordinated by oxygen atoms due to their small ionic radii.

### Experimental

The composition of the mixture for crystal growth was 10:1:10 of Na_2_CO_3_ (analytically pure), Sc_2_O_3_ (analytically pure), and B_2_O_3_ (analytically pure). This mixture was heated in a platinum crucible to 1373 K, held at this temperature for several hours, and then the transparent melt was cooled slowly from 1373 K to 1223 K at 3 K h^-1^. Upon further cooling to room temperature, block shaped colorless crystals were obtained.

### Refinement

The structure was solved with the direct methods program *SHELXS97* and refined with the least-squares program *SHELXL97* of the *SHELXTL*.PC suite of programs. The final refinement included anisotropic displacement parameters and a secondary extinction correction. The program *STRUCTURE TIDY* (Gelato & Parthé, 1987) was then employed to standardize the atomic coordinates.

### Crystal data

**Table:**

Na_3_Sc(BO_3_)_2_	*F*(000) = 224
*M**_r_* = 231.55	*D*_x_ = 2.879 Mg m^−^^3^
Monoclinic, *P*2_1_/*c*	Mo *K*α radiation, λ = 0.71073 Å
Hall symbol: -P 2ybc	Cell parameters from 2093 reflections
*a* = 5.0739 (10) Å	θ = 3.6–31.0°
*b* = 8.9930 (18) Å	µ = 1.58 mm^−^^1^
*c* = 7.029 (2) Å	*T* = 153 K
β = 123.60 (2)°	Prism, colourless
*V* = 267.14 (11) Å^3^	0.27 × 0.14 × 0.09 mm
*Z* = 2	

### Data collection

**Table:**

Rigaku Saturn724+ diffractometer	847 independent reflections
Radiation source: fine-focus sealed tube	760 reflections with *I* > 2σ(*I*)
Graphite monochromator	*R*_int_ = 0.026
Detector resolution: 28.5714 pixels mm^-1^	θ_max_ = 30.9°, θ_min_ = 4.2°
ω scans	*h* = −7→6
Absorption correction: multi-scan (*ABSCOR*; Higashi, 1995)	*k* = −13→13
*T*_min_ = 0.824, *T*_max_ = 1.000	*l* = −10→8
3029 measured reflections	

### Refinement

**Table:**

Refinement on *F*^2^	0 restraints
Least-squares matrix: full	Primary atom site location: structure-invariant direct methods
*R*[*F*^2^ > 2σ(*F*^2^)] = 0.027	Secondary atom site location: difference Fourier map
*wR*(*F*^2^) = 0.067	*w* = 1/[σ^2^(*F*_o_^2^) + (0.030*P*)^2^] where *P* = (*F*_o_^2^ + 2*F*_c_^2^)/3
*S* = 1.22	(Δ/σ)_max_ < 0.001
847 reflections	Δρ_max_ = 0.35 e Å^−^^3^
58 parameters	Δρ_min_ = −0.62 e Å^−^^3^

### Special details

**Table:**

Geometry. All e.s.d.'s (except the e.s.d. in the dihedral angle between two l.s. planes) are estimated using the full covariance matrix. The cell e.s.d.'s are taken into account individually in the estimation of e.s.d.'s in distances, angles and torsion angles; correlations between e.s.d.'s in cell parameters are only used when they are defined by crystal symmetry. An approximate (isotropic) treatment of cell e.s.d.'s is used for estimating e.s.d.'s involving l.s. planes.
Refinement. Refinement of *F*^2^ against ALL reflections. The weighted *R*-factor *wR* and goodness of fit *S* are based on *F*^2^, conventional *R*-factors *R* are based on *F*, with *F* set to zero for negative *F*^2^. The threshold expression of *F*^2^ > σ(*F*^2^) is used only for calculating *R*-factors(gt) *etc*. and is not relevant to the choice of reflections for refinement. *R*-factors based on *F*^2^ are statistically about twice as large as those based on *F*, and *R*- factors based on ALL data will be even larger.

### Fractional atomic coordinates and isotropic or equivalent isotropic displacement parameters (Å^2^)

**Table:**

	*x*	*y*	*z*	*U*_iso_*/*U*_eq_	
Sc1	0.5000	0.0000	0.0000	0.00372 (11)	
O1	0.4755 (2)	0.08396 (12)	0.27296 (17)	0.0073 (2)	
O2	−0.0650 (2)	0.10637 (11)	0.15507 (18)	0.0076 (2)	
O3	0.2676 (2)	0.31781 (12)	0.29120 (18)	0.0086 (2)	
B1	0.2264 (4)	0.16821 (18)	0.2386 (2)	0.0052 (3)	
Na1	1.0000	0.0000	0.5000	0.00806 (18)	
Na2	0.73774 (13)	0.33234 (8)	0.23583 (10)	0.01153 (16)	

### Atomic displacement parameters (Å^2^)

**Table:**

	*U*^11^	*U*^22^	*U*^33^	*U*^12^	*U*^13^	*U*^23^
Sc1	0.00282 (19)	0.00312 (18)	0.00441 (18)	−0.00007 (12)	0.00150 (14)	−0.00011 (12)
O1	0.0057 (5)	0.0083 (5)	0.0072 (5)	0.0011 (4)	0.0030 (4)	−0.0006 (4)
O2	0.0054 (5)	0.0077 (5)	0.0087 (5)	−0.0022 (4)	0.0033 (4)	−0.0023 (4)
O3	0.0084 (5)	0.0047 (5)	0.0104 (5)	−0.0016 (4)	0.0038 (4)	−0.0021 (4)
B1	0.0066 (7)	0.0054 (7)	0.0039 (7)	−0.0007 (5)	0.0032 (6)	−0.0007 (5)
Na1	0.0061 (4)	0.0092 (4)	0.0065 (4)	0.0002 (3)	0.0020 (3)	−0.0004 (3)
Na2	0.0100 (3)	0.0111 (3)	0.0143 (3)	0.0014 (2)	0.0072 (3)	−0.0017 (2)

### Geometric parameters (Å, º)

**Table:**

Sc1—O2^i^	2.0765 (11)	O3—Na2	2.6242 (12)
Sc1—O2^ii^	2.0765 (11)	O3—Na2^vii^	3.0050 (15)
Sc1—O3^iii^	2.0769 (11)	B1—Na2^viii^	2.8761 (17)
Sc1—O3^iv^	2.0769 (11)	B1—Na2^vii^	2.9851 (19)
Sc1—O1	2.1285 (11)	B1—Na2^ix^	2.9897 (19)
Sc1—O1^v^	2.1285 (11)	B1—Na2	2.9945 (17)
Sc1—Na1^vi^	2.9866 (11)	B1—Na2^iv^	3.0253 (19)
Sc1—Na1	2.9866 (11)	B1—Na1^viii^	3.0528 (16)
Sc1—Na2^iv^	3.1073 (8)	Na1—O1^xi^	2.3448 (12)
Sc1—Na2^iii^	3.1073 (8)	Na1—O3^iv^	2.3752 (12)
Sc1—Na2^v^	3.3049 (9)	Na1—O3^xii^	2.3752 (12)
Sc1—Na2	3.3049 (9)	Na1—O2^xiii^	2.4529 (12)
O1—B1	1.3758 (17)	Na1—O2^ii^	2.4529 (12)
O1—Na1	2.3448 (12)	Na1—Sc1^xiv^	2.9866 (11)
O1—Na2^iv^	2.4942 (13)	Na1—Na2^vii^	3.0386 (8)
O1—Na2	2.6855 (12)	Na1—Na2^xv^	3.0386 (8)
O1—Na2^vii^	2.8515 (15)	Na1—B1^xiii^	3.0528 (16)
O2—B1	1.3708 (17)	Na1—B1^ii^	3.0528 (16)
O2—Sc1^viii^	2.0765 (11)	Na2—O2^ii^	2.4662 (12)
O2—Na1^viii^	2.4529 (12)	Na2—O1^x^	2.4942 (13)
O2—Na2^viii^	2.4662 (12)	Na2—O3^ii^	2.4976 (12)
O2—Na2^ix^	2.5933 (14)	Na2—O2^xii^	2.5933 (14)
O2—Na2^iv^	2.8301 (13)	Na2—O2^x^	2.8301 (13)
O3—B1	1.3802 (19)	Na2—O1^iii^	2.8515 (15)
O3—Sc1^x^	2.0769 (11)	Na2—B1^ii^	2.8761 (17)
O3—Na1^x^	2.3752 (12)	Na2—B1^iii^	2.985 (2)
O3—Na2^viii^	2.4976 (12)	Na2—B1^xii^	2.990 (2)
			
O2^i^—Sc1—O2^ii^	180.00 (5)	O1—B1—Na1^viii^	103.69 (9)
O2^i^—Sc1—O3^iii^	88.26 (4)	O3—B1—Na1^viii^	111.93 (9)
O2^ii^—Sc1—O3^iii^	91.74 (4)	Na2^viii^—B1—Na1^viii^	69.41 (4)
O2^i^—Sc1—O3^iv^	91.74 (4)	Na2^vii^—B1—Na1^viii^	70.56 (4)
O2^ii^—Sc1—O3^iv^	88.26 (4)	Na2^ix^—B1—Na1^viii^	111.63 (5)
O3^iii^—Sc1—O3^iv^	180.00 (4)	Na2—B1—Na1^viii^	149.78 (6)
O2^i^—Sc1—O1	93.52 (4)	Na2^iv^—B1—Na1^viii^	59.99 (3)
O2^ii^—Sc1—O1	86.48 (4)	O1—Na1—O1^xi^	180.0
O3^iii^—Sc1—O1	93.65 (4)	O1—Na1—O3^iv^	75.13 (4)
O3^iv^—Sc1—O1	86.35 (4)	O1^xi^—Na1—O3^iv^	104.87 (4)
O2^i^—Sc1—O1^v^	86.48 (4)	O1—Na1—O3^xii^	104.87 (4)
O2^ii^—Sc1—O1^v^	93.52 (4)	O1^xi^—Na1—O3^xii^	75.13 (4)
O3^iii^—Sc1—O1^v^	86.35 (4)	O3^iv^—Na1—O3^xii^	180.0
O3^iv^—Sc1—O1^v^	93.65 (4)	O1—Na1—O2^xiii^	106.23 (4)
O1—Sc1—O1^v^	180.00 (2)	O1^xi^—Na1—O2^xiii^	73.77 (4)
O2^i^—Sc1—Na1^vi^	54.43 (3)	O3^iv^—Na1—O2^xiii^	106.43 (4)
O2^ii^—Sc1—Na1^vi^	125.57 (3)	O3^xii^—Na1—O2^xiii^	73.57 (4)
O3^iii^—Sc1—Na1^vi^	52.27 (3)	O1—Na1—O2^ii^	73.77 (4)
O3^iv^—Sc1—Na1^vi^	127.73 (3)	O1^xi^—Na1—O2^ii^	106.23 (4)
O1—Sc1—Na1^vi^	128.72 (3)	O3^iv^—Na1—O2^ii^	73.57 (4)
O1^v^—Sc1—Na1^vi^	51.28 (3)	O3^xii^—Na1—O2^ii^	106.43 (4)
O2^i^—Sc1—Na1	125.57 (3)	O2^xiii^—Na1—O2^ii^	180.0
O2^ii^—Sc1—Na1	54.43 (3)	O1—Na1—Sc1^xiv^	134.91 (3)
O3^iii^—Sc1—Na1	127.73 (3)	O1^xi^—Na1—Sc1^xiv^	45.09 (3)
O3^iv^—Sc1—Na1	52.27 (3)	O3^iv^—Na1—Sc1^xiv^	136.25 (3)
O1—Sc1—Na1	51.28 (3)	O3^xii^—Na1—Sc1^xiv^	43.75 (3)
O1^v^—Sc1—Na1	128.72 (3)	O2^xiii^—Na1—Sc1^xiv^	43.52 (3)
Na1^vi^—Sc1—Na1	180.0	O2^ii^—Na1—Sc1^xiv^	136.48 (3)
O2^i^—Sc1—Na2^iv^	55.86 (4)	O1—Na1—Sc1	45.09 (3)
O2^ii^—Sc1—Na2^iv^	124.14 (3)	O1^xi^—Na1—Sc1	134.91 (3)
O3^iii^—Sc1—Na2^iv^	123.28 (3)	O3^iv^—Na1—Sc1	43.75 (3)
O3^iv^—Sc1—Na2^iv^	56.72 (3)	O3^xii^—Na1—Sc1	136.25 (3)
O1—Sc1—Na2^iv^	52.98 (3)	O2^xiii^—Na1—Sc1	136.48 (3)
O1^v^—Sc1—Na2^iv^	127.02 (3)	O2^ii^—Na1—Sc1	43.52 (3)
Na1^vi^—Sc1—Na2^iv^	110.20 (2)	Sc1^xiv^—Na1—Sc1	180.0
Na1—Sc1—Na2^iv^	69.80 (2)	O1—Na1—Na2^vii^	62.41 (3)
O2^i^—Sc1—Na2^iii^	124.14 (4)	O1^xi^—Na1—Na2^vii^	117.59 (3)
O2^ii^—Sc1—Na2^iii^	55.86 (4)	O3^iv^—Na1—Na2^vii^	126.75 (3)
O3^iii^—Sc1—Na2^iii^	56.72 (3)	O3^xii^—Na1—Na2^vii^	53.25 (3)
O3^iv^—Sc1—Na2^iii^	123.28 (3)	O2^xiii^—Na1—Na2^vii^	60.94 (3)
O1—Sc1—Na2^iii^	127.02 (3)	O2^ii^—Na1—Na2^vii^	119.06 (3)
O1^v^—Sc1—Na2^iii^	52.98 (3)	Sc1^xiv^—Na1—Na2^vii^	72.62 (2)
Na1^vi^—Sc1—Na2^iii^	69.80 (2)	Sc1—Na1—Na2^vii^	107.38 (2)
Na1—Sc1—Na2^iii^	110.20 (2)	O1—Na1—Na2^xv^	117.59 (3)
Na2^iv^—Sc1—Na2^iii^	180.00 (3)	O1^xi^—Na1—Na2^xv^	62.41 (3)
O2^i^—Sc1—Na2^v^	48.18 (3)	O3^iv^—Na1—Na2^xv^	53.25 (3)
O2^ii^—Sc1—Na2^v^	131.82 (3)	O3^xii^—Na1—Na2^xv^	126.75 (3)
O3^iii^—Sc1—Na2^v^	116.88 (4)	O2^xiii^—Na1—Na2^xv^	119.06 (3)
O3^iv^—Sc1—Na2^v^	63.12 (4)	O2^ii^—Na1—Na2^xv^	60.94 (3)
O1—Sc1—Na2^v^	125.86 (3)	Sc1^xiv^—Na1—Na2^xv^	107.38 (2)
O1^v^—Sc1—Na2^v^	54.14 (3)	Sc1—Na1—Na2^xv^	72.62 (2)
Na1^vi^—Sc1—Na2^v^	64.735 (14)	Na2^vii^—Na1—Na2^xv^	180.0
Na1—Sc1—Na2^v^	115.265 (14)	O1—Na1—B1^xiii^	84.60 (4)
Na2^iv^—Sc1—Na2^v^	72.919 (18)	O1^xi^—Na1—B1^xiii^	95.40 (4)
Na2^iii^—Sc1—Na2^v^	107.081 (18)	O3^iv^—Na1—B1^xiii^	87.18 (4)
O2^i^—Sc1—Na2	131.82 (3)	O3^xii^—Na1—B1^xiii^	92.82 (4)
O2^ii^—Sc1—Na2	48.18 (3)	O2^xiii^—Na1—B1^xiii^	26.03 (4)
O3^iii^—Sc1—Na2	63.12 (4)	O2^ii^—Na1—B1^xiii^	153.97 (4)
O3^iv^—Sc1—Na2	116.88 (4)	Sc1^xiv^—Na1—B1^xiii^	69.47 (3)
O1—Sc1—Na2	54.14 (3)	Sc1—Na1—B1^xiii^	110.53 (3)
O1^v^—Sc1—Na2	125.86 (3)	Na2^vii^—Na1—B1^xiii^	59.56 (4)
Na1^vi^—Sc1—Na2	115.265 (14)	Na2^xv^—Na1—B1^xiii^	120.44 (4)
Na1—Sc1—Na2	64.735 (14)	O1—Na1—B1^ii^	95.40 (4)
Na2^iv^—Sc1—Na2	107.081 (18)	O1^xi^—Na1—B1^ii^	84.60 (4)
Na2^iii^—Sc1—Na2	72.919 (18)	O3^iv^—Na1—B1^ii^	92.82 (4)
Na2^v^—Sc1—Na2	180.00 (2)	O3^xii^—Na1—B1^ii^	87.18 (4)
B1—O1—Sc1	122.92 (9)	O2^xiii^—Na1—B1^ii^	153.97 (4)
B1—O1—Na1	152.05 (9)	O2^ii^—Na1—B1^ii^	26.03 (4)
Sc1—O1—Na1	83.62 (4)	Sc1^xiv^—Na1—B1^ii^	110.53 (3)
B1—O1—Na2^iv^	98.70 (8)	Sc1—Na1—B1^ii^	69.47 (3)
Sc1—O1—Na2^iv^	84.07 (4)	Na2^vii^—Na1—B1^ii^	120.44 (4)
Na1—O1—Na2^iv^	92.19 (4)	Na2^xv^—Na1—B1^ii^	59.56 (4)
B1—O1—Na2	88.93 (8)	B1^xiii^—Na1—B1^ii^	180.0
Sc1—O1—Na2	85.89 (4)	O2^ii^—Na2—O1^x^	165.38 (4)
Na1—O1—Na2	84.11 (4)	O2^ii^—Na2—O3^ii^	56.90 (4)
Na2^iv^—O1—Na2	169.63 (5)	O1^x^—Na2—O3^ii^	117.78 (4)
B1—O1—Na2^vii^	81.85 (8)	O2^ii^—Na2—O2^xii^	119.08 (4)
Sc1—O1—Na2^vii^	154.03 (5)	O1^x^—Na2—O2^xii^	74.02 (4)
Na1—O1—Na2^vii^	70.81 (4)	O3^ii^—Na2—O2^xii^	97.40 (4)
Na2^iv^—O1—Na2^vii^	100.94 (4)	O2^ii^—Na2—O3	120.74 (4)
Na2—O1—Na2^vii^	87.01 (4)	O1^x^—Na2—O3	68.37 (4)
B1—O2—Sc1^viii^	173.15 (9)	O3^ii^—Na2—O3	164.30 (6)
B1—O2—Na1^viii^	102.22 (8)	O2^xii^—Na2—O3	69.69 (4)
Sc1^viii^—O2—Na1^viii^	82.05 (4)	O2^ii^—Na2—O1	67.85 (4)
B1—O2—Na2^viii^	92.64 (8)	O1^x^—Na2—O1	121.70 (2)
Sc1^viii^—O2—Na2^viii^	92.96 (4)	O3^ii^—Na2—O1	119.33 (4)
Na1^viii^—O2—Na2^viii^	86.77 (4)	O2^xii^—Na2—O1	88.09 (4)
B1—O2—Na2^ix^	92.69 (8)	O3—Na2—O1	53.44 (3)
Sc1^viii^—O2—Na2^ix^	82.63 (4)	O2^ii^—Na2—O2^x^	121.86 (2)
Na1^viii^—O2—Na2^ix^	164.22 (5)	O1^x^—Na2—O2^x^	53.00 (4)
Na2^viii^—O2—Na2^ix^	97.81 (4)	O3^ii^—Na2—O2^x^	65.40 (4)
B1—O2—Na2^iv^	84.56 (8)	O2^xii^—Na2—O2^x^	72.99 (4)
Sc1^viii^—O2—Na2^iv^	92.00 (4)	O3—Na2—O2^x^	116.64 (4)
Na1^viii^—O2—Na2^iv^	69.81 (3)	O1—Na2—O2^x^	161.07 (4)
Na2^viii^—O2—Na2^iv^	155.11 (4)	O2^ii^—Na2—O1^iii^	86.99 (4)
Na2^ix^—O2—Na2^iv^	107.01 (4)	O1^x^—Na2—O1^iii^	79.06 (4)
B1—O3—Sc1^x^	154.28 (10)	O3^ii^—Na2—O1^iii^	88.51 (4)
B1—O3—Na1^x^	120.79 (9)	O2^xii^—Na2—O1^iii^	152.06 (5)
Sc1^x^—O3—Na1^x^	83.98 (4)	O3—Na2—O1^iii^	107.09 (4)
B1—O3—Na2^viii^	91.07 (8)	O1—Na2—O1^iii^	112.77 (4)
Sc1^x^—O3—Na2^viii^	102.13 (4)	O2^x^—Na2—O1^iii^	84.99 (4)
Na1^x^—O3—Na2^viii^	77.11 (4)	O2^ii^—Na2—B1^ii^	28.43 (4)
B1—O3—Na2	91.39 (8)	O1^x^—Na2—B1^ii^	145.72 (5)
Sc1^x^—O3—Na2	81.85 (4)	O3^ii^—Na2—B1^ii^	28.67 (4)
Na1^x^—O3—Na2	88.33 (4)	O2^xii^—Na2—B1^ii^	108.19 (5)
Na2^viii^—O3—Na2	164.30 (6)	O3—Na2—B1^ii^	145.52 (5)
B1—O3—Na2^vii^	75.88 (8)	O1—Na2—B1^ii^	92.52 (5)
Sc1^x^—O3—Na2^vii^	78.82 (4)	O2^x^—Na2—B1^ii^	94.05 (4)
Na1^x^—O3—Na2^vii^	162.26 (5)	O1^iii^—Na2—B1^ii^	89.96 (4)
Na2^viii^—O3—Na2^vii^	110.62 (5)	O2^ii^—Na2—B1^iii^	85.97 (4)
Na2—O3—Na2^vii^	85.01 (4)	O1^x^—Na2—B1^iii^	83.59 (4)
O2—B1—O1	121.35 (13)	O3^ii^—Na2—B1^iii^	110.22 (5)
O2—B1—O3	118.55 (12)	O2^xii^—Na2—B1^iii^	150.41 (4)
O1—B1—O3	120.10 (12)	O3—Na2—B1^iii^	84.32 (5)
O2—B1—Na2^viii^	58.93 (7)	O1—Na2—B1^iii^	87.48 (4)
O1—B1—Na2^viii^	171.39 (10)	O2^x^—Na2—B1^iii^	108.55 (4)
O3—B1—Na2^viii^	60.25 (7)	O1^iii^—Na2—B1^iii^	27.14 (4)
O2—B1—Na2^vii^	122.23 (9)	B1^ii^—Na2—B1^iii^	101.22 (5)
O1—B1—Na2^vii^	71.01 (8)	O2^ii^—Na2—B1^xii^	95.39 (4)
O3—B1—Na2^vii^	77.48 (8)	O1^x^—Na2—B1^xii^	95.47 (4)
Na2^viii^—B1—Na2^vii^	101.32 (5)	O3^ii^—Na2—B1^xii^	72.47 (5)
O2—B1—Na2^ix^	60.05 (7)	O2^xii^—Na2—B1^xii^	27.26 (4)
O1—B1—Na2^ix^	106.68 (9)	O3—Na2—B1^xii^	92.97 (5)
O3—B1—Na2^ix^	102.82 (9)	O1—Na2—B1^xii^	90.87 (4)
Na2^viii^—B1—Na2^ix^	81.08 (5)	O2^x^—Na2—B1^xii^	72.71 (4)
Na2^vii^—B1—Na2^ix^	177.30 (6)	O1^iii^—Na2—B1^xii^	155.12 (4)
O2—B1—Na2	158.47 (10)	B1^ii^—Na2—B1^xii^	80.99 (5)
O1—B1—Na2	63.72 (7)	B1^iii^—Na2—B1^xii^	177.30 (6)
O3—B1—Na2	61.17 (7)	O2^ii^—Na2—B1	93.37 (5)
Na2^viii^—B1—Na2	119.59 (6)	O1^x^—Na2—B1	94.69 (4)
Na2^vii^—B1—Na2	79.25 (4)	O3^ii^—Na2—B1	146.62 (5)
Na2^ix^—B1—Na2	98.50 (5)	O2^xii^—Na2—B1	83.59 (4)
O2—B1—Na2^iv^	68.63 (8)	O3—Na2—B1	27.44 (4)
O1—B1—Na2^iv^	54.58 (7)	O1—Na2—B1	27.35 (4)
O3—B1—Na2^iv^	164.17 (10)	O2^x^—Na2—B1	143.91 (4)
Na2^viii^—B1—Na2^iv^	122.42 (5)	O1^iii^—Na2—B1	106.18 (4)
Na2^vii^—B1—Na2^iv^	86.79 (4)	B1^ii^—Na2—B1	119.59 (6)
Na2^ix^—B1—Na2^iv^	92.97 (5)	B1^iii^—Na2—B1	79.16 (5)
Na2—B1—Na2^iv^	117.94 (5)	B1^xii^—Na2—B1	98.41 (5)
O2—B1—Na1^viii^	51.75 (7)		

Symmetry codes: (i) −*x*, −*y*, −*z*; (ii) *x*+1, *y*, *z*; (iii) *x*, −*y*+1/2, *z*−1/2; (iv) −*x*+1, *y*−1/2, −*z*+1/2; (v) −*x*+1, −*y*, −*z*; (vi) *x*−1, *y*, *z*−1; (vii) *x*, −*y*+1/2, *z*+1/2; (viii) *x*−1, *y*, *z*; (ix) *x*−1, −*y*+1/2, *z*−1/2; (x) −*x*+1, *y*+1/2, −*z*+1/2; (xi) −*x*+2, −*y*, −*z*+1; (xii) *x*+1, −*y*+1/2, *z*+1/2; (xiii) −*x*+1, −*y*, −*z*+1; (xiv) *x*+1, *y*, *z*+1; (xv) −*x*+2, *y*−1/2, −*z*+1/2.
